# Amikacin liposome inhalation suspension clinical benefit–risk assessment for refractory *Mycobacterium avium* complex lung disease

**DOI:** 10.1183/23120541.00623-2021

**Published:** 2022-07-11

**Authors:** Theodore K. Marras, Mariam Hassan, Kevin C. Mange, Monika Ciesielska, Shilpa Dhar Murthy, Zhanna Jumadilova, Anjan Chatterjee

**Affiliations:** 1 Dept of Medicine, University of Toronto and Toronto Western Hospital, Toronto, ON, Canada; 2 Insmed Incorporated, Bridgewater, NJ, USA

## Abstract

*Mycobacterium avium* complex (MAC) is the leading cause of nontuberculous mycobacterial lung disease, which can be associated with progressive lung damage and increased mortality [1]. Patients with MAC lung disease have substantial disease burden and limited treatment options [1]. Up to 40% of patients experience failure, with lengthy multidrug treatments, relapse or reinfection [2]. For patients with treatment-refractory MAC lung disease (persistent MAC-positive sputum despite ≥6 months of guideline-based therapy (GBT)), international guidelines recommend the addition of amikacin liposome inhalation suspension (ALIS) to GBT regimens [3]. In clinical trials, patients with treatment-refractory MAC lung disease had improved culture conversion with ALIS+GBT *versus* GBT [4, 5].

## Abstract 2

**Marras *et al*. report a low number needed to treat and high number needed to harm supporting addition of amikacin liposome inhalation suspension to guideline-based treatments in patients with treatment-refractory *Mycobacterium avium* complex lung disease** https://bit.ly/3tPFW7D

*To the Editor:*

*Mycobacterium avium* complex (MAC) is the leading cause of nontuberculous mycobacterial lung disease, which can be associated with progressive lung damage and increased mortality [1]. Patients with MAC lung disease have substantial disease burden and limited treatment options [1]. Up to 40% of patients experience failure, with lengthy multidrug treatments, relapse or reinfection [2]. For patients with treatment-refractory MAC lung disease (persistent MAC-positive sputum despite ≥6 months of guideline-based therapy (GBT)), international guidelines recommend the addition of amikacin liposome inhalation suspension (ALIS) to GBT regimens [3]. In clinical trials, patients with treatment-refractory MAC lung disease had improved culture conversion with ALIS+GBT *versus* GBT [4, 5].

To facilitate benefit and risk interpretation for clinical care, the number needed to treat (NNT) and number needed to harm (NNH) values indicate how many patients would need to receive treatment over a comparator until one patient experienced that benefit or risk, respectively [6]. These *post hoc* analyses of ALIS clinical trial data aimed to assess the NNTs and NNHs for ALIS+GBT compared with GBT in patients with treatment-refractory MAC lung disease.

We studied results from adults with confirmed MAC lung disease diagnoses [7] and persistently positive sputum cultures despite ≥6 months of GBT who were enrolled in clinical trials evaluating the efficacy and safety of adding ALIS (or placebo) to continued GBT [5]. Our analyses included the phase 3, open-label, randomised (2:1) CONVERT study (www.clinicaltrials.gov identifier number NCT02344004) [5, 8], an open-label safety extension of CONVERT (study INS-312; NCT02628600) [8, 9], and a phase 2, double-blind, placebo-controlled study with an open-label extension (study TR02–112; NCT01315236) [4, 9]. Patient inclusion and exclusion criteria for these studies have been reported [4, 5, 8, 9]. Study protocols and patient informed consent forms were reviewed and approved by an independent ethics committee or institutional review board at each site in these studies [4, 5, 8, 9].

The benefit of ALIS over GBT was assessed using CONVERT data (ALIS+GBT (n=224) *versus* GBT alone (n=112)) [5, 8]. NNTs were calculated for culture conversion by month 6, sustained culture conversion by month 12 and durable culture conversion 3 months off all MAC treatments in patients completing 12 months of post-conversion treatment [6]. For the risk of adverse events of special interest with ALIS compared with GBT alone or with placebo, pooled data from all three clinical trials (ALIS+GBT (n=404) *versus* GBT±placebo (n=157)) [4, 5, 8, 9] were assessed, including ototoxicity, nephrotoxicity, neuromuscular effects and allergic alveolitis. Due to varied durations of treatment across studies, unadjusted and exposure-adjusted NNH values were calculated [6]. The risk difference between treatment arms was calculated with 95% confidence intervals for NNT and NNH. When the two-sided 95% confidence interval for the risk difference included 0, as may occur in the case of rare events in very small populations, the 95% confidence interval for NNH included infinity. In these cases, the worst-case scenario for the lower bound of the NNH has been reported. The upper bounds of the 95% confidence intervals for most reported NNH values were infinite (*i.e*. an infinite number of patients would be required to show any harm within the 95% CI).

The NNTs were determined from results in the CONVERT study (N=336) and are presented in table 1 [8]. In patients achieving culture conversion by month 6 of treatment, the NNT was 5 (95% CI 3.6–8.2) with 29.0% of patients achieving culture conversion when treated with ALIS+GBT *versus* 8.9% of patients treated with GBT alone. At 12 months of sustained conversion, the NNT was 6 (95% CI 4.6–10.3) and a higher proportion of patients had sustained culture conversion with ALIS+GBT *versus* GBT alone (18.3% *versus* 2.7%). For durable culture conversion at 3 months off all MAC treatments, the NNT was 6 (95% CI 4.8–8.9) and a higher proportion of patients had durable culture conversion with ALIS+GBT *versus* GBT alone (16.1% *versus* 0%).

**TABLE 1:** Number needed to treat (NNT) and number needed to harm (NNH) for amikacin liposome inhalation suspension (ALIS) plus guideline-based therapy (GBT) *versus* GBT±placebo

**NNT for ALIS+GBT *versus* GBT alone in the CONVERT trial and safety extension [5, 8]**	**ALIS+GBT (n=224), n (%)**	**GBT (n=112), n (%)**	**NNT (95% CI)**
**Culture conversion by month 6**	65 (29.0)	10 (8.9)	5 (3.6–8.2)
**Sustained conversion at 12 months of treatment**	41 (18.3)^#^	3 (2.7)	6 (4.6–10.3)
**Durable conversion at follow-up 3 months off treatment**	36 (16.1)^¶^	0	6 (4.8–8.9)
**NNH for ALIS+GBT *versus* GBT±placebo in a pooled safety population [4, 5, 8, 9]**	**ALIS+GBT (n=404), n (%)**	**GBT±placebo (n=157), n (%)**	**NNH (95% CI)**
**Unadjusted**			
Ototoxicity	72 (17.8)	16 (10.2)	13 (7.3–62.3)
Nephrotoxicity	17 (4.2)	4 (2.5)	60 (>20.8^ƒ^)
Neuromuscular effects	12 (3.0)	1 (0.6)	43 (22.7–381.1)
Allergic alveolitis	13 (3.2)	2 (1.3)	51 (>22.7^ƒ^)
	**ALIS+GBT (327 patient-years), n (%)** ^§^	**GBT±placebo (87 patient-years), n (%)** ^§^	**NNH (95% CI)**
**Exposure-adjusted^+^**			
Ototoxicity	72 (22.0)	16 (18.4)	28 (>7.7^ƒ^)
Nephrotoxicity	17 (5.2)	4 (4.6)	166 (>17.8^ƒ^)
Neuromuscular effects	12 (3.7)	1 (1.1)	40 (>18.0^ƒ^)
Allergic alveolitis	13 (4.0)	2 (2.3)	60 (>18.3^ƒ^)

^#^: at 12 months of treatment, five patients experienced relapse of *Mycobacterium avium* complex (MAC) lung disease with the same species and strain, three patients had reinfection with a different MAC species or strain, and 16 patients had no sputum data at this time point [8]. ^¶^: at 3 months off treatment, one additional patient experienced relapse of MAC lung disease with the same species and strain, and four patients had no sputum data at this time point [8]. ^+^: adjusted for difference in exposure time to ALIS *versus* GBT. ^§^: incidence rate per 100 patient-years was calculated as (number of patients with events/total exposure in years)×100. ^*f*^: the two-sided 95% confidence interval of risk difference includes 0; therefore, noncontinuous 95% confidence intervals are generated when the upper bounds of the 95% confidence interval are infinite. The lower bound of NNH (unadjusted) was 20.8 for nephrotoxicity and 22.7 for allergic alveolitis; for NNH (adjusted), the lower bounds were 7.7 for ototoxicity, 17.8 for nephrotoxicity, 18.0 for neuromuscular events and 18.3 for allergic alveolitis.

Risk estimates of adverse events of special interest with ALIS were determined using pooled safety data from all three clinical trials (N=561) [4, 5, 8, 9]. The unadjusted NNH values were 13 for ototoxicity, 60 for nephrotoxicity, 43 for neuromuscular effects and 51 for allergic alveolitis (table 1). Exposure-adjusted NNHs were calculated to account for differences in treatment durations across studies (327 patient-years for ALIS+GBT; 87 patient-years for GBT±placebo). The exposure-adjusted NNHs were 28 for ototoxicity, 166 for nephrotoxicity, 40 for neuromuscular effects and 60 for allergic alveolitis (table 1). Ototoxicity was reported in 72 out of 404 patients treated with ALIS+GBT (17.8%), mainly comprising tinnitus (6.9%) and dizziness (5.9%). Other ototoxicity symptoms (deafness, deafness neurosensory, deafness unilateral, hypoacusis, balance disorder, presyncope and vertigo) were each reported in <3% of patients treated with ALIS+GBT. The exposure-adjusted NNHs for ototoxicity symptoms were 20 for tinnitus, 36 for dizziness and >40 for other ototoxicity symptoms (data not shown).

In evaluating the benefit and risk of a treatment, NNT and NNH measures help clinicians intuit statistical data to understand how treatments can impact specific numbers of patients and how clinical trial data relate to real-world practice [6]. This report highlights the substantial benefits observed in the phase 3 CONVERT study and safety extension due to a low NNT for culture conversion in the ALIS+GBT group (*versus* the GBT-alone group) (NNT 5 by 6 months of treatment). In addition, the low NNTs for sustained conversion at 12 months of post-conversion treatment (NNT 6) and durable conversion at 3 months off all MAC treatment (NNT 6) support the long-term benefit of ALIS+GBT over GBT alone. Because NNTs are typically higher in difficult-to-treat populations and when active comparators are compared with placebo, the low NNTs reported here for the addition of ALIS to a multidrug regimen are particularly notable [10].

Unadjusted NNHs for adverse events of special interest ranged from 13 for ototoxicity to 60 for nephrotoxicity. When NNH values were exposure-adjusted for the greater treatment duration with ALIS+GBT *versus* GBT across studies (327 *versus* 87 patient-years), exposure-adjusted NNHs ranged from 28 for ototoxicity to 166 for nephrotoxicity. The lowest NNH was for ototoxicity, and was largely driven by tinnitus and dizziness. The low single-digit NNT and higher NNH values observed in these analyses support the favourable safety profile of ALIS+GBT in offering a clinically meaningful treatment for patients with treatment-refractory MAC lung disease.
